# COVID-19 provides an opportunity to build a resilient and equitable immunization delivery system

**DOI:** 10.11604/pamj.supp.2022.41.2.28420

**Published:** 2022-03-29

**Authors:** Antoinette Eleonore Ba-Nguz, Mohamed Diaaeldin Omer, Niklas Danielsson, Flint Zulu, Dereje Haile

**Affiliations:** 1 UNICEF Regional Office for Eastern and Southern Africa, Kenya

**Keywords:** COVID-19, immunization, resilient system, health system

## Abstract

The COVID-19 pandemic has weakened the health systems in many countries particularly putting at risk efforts on the Immunization Agenda to make vaccination available to everyone, everywhere, by 2030. Immunization Agenda 2030 reconfirms the importance of reducing the absolute number of zero-dose children and increasing the proportion of children who complete their vaccinations on time. Despite the gains in promoting equity in immunization services, many children missed vaccination because of the COVID-19 pandemic that disrupted well planned strategies. The cancellation of outreach services following the COVID-19-motivated lockdown meant many children missed vaccination. The situation was further worsened by vaccination related rumors and fears. The collapse of the Primary Health Care (PHC) service provision during the epidemic may lead to higher under-five mortality similar to the Ebola Virus Disease epidemic in West Africa. The post COVID-19 recovery strategy should include strengthening the service delivery systems to remain resilient when threatened by emergencies. The recovery must therefore focus on rebuilding trust as the foundation for vaccine acceptance and demand which can only be achieved by appropriate community engagement.

## Opinion

African children are at the highest risk of dying from vaccine preventable diseases in the world. The pandemic disrupted public health and social services across the continent and efforts to restore routine immunization services have already started. The COVID-19 pandemic presents an opportunity to build a better and more equitable immunization service delivery system that will close the yawning vaccination gap in sub-Saharan Africa. The public support and political will for vaccination is an opportunity for sub-Saharan African (SSA) countries to close the immunization equity gap that leaves more than 6 million children unvaccinated each year in the region. African leaders, ministers of health, UN organizations and national and international immunization partners must make the most of this unique chance to ensure that the right of every child born in region to be protected against vaccine preventable diseases is realized during the IA2030 decade. It is time to start planning beyond the end of the COVID-19 pandemic for a more resilient, fair and effective immunization and primary health care services.

### COVID-19 pandemic has limited access to immunization

Immunization is acknowledged as probably the most successful public health intervention [1]. Countries have made notable efforts over the last decades to protect their children from potentially deadly infections. Over the last two years, the African Region has made the highest gain in routine vaccination coverage, with 25% points increase (from 50% to 74%) in the third dose of diphtheria, tetanus and pertussis- containing vaccine (DTP-3). Nonetheless, the region hosts almost 50% of the 20 million children who miss their DTP3 dose globally each year. Close to seven million children are not vaccinated [2]). Children that miss vaccines are also likely to miss other basic health services.

In 2020 various partners meetings, countries reported substantial reduction in coverage: relative difference in DTP3 coverage of around 15% between January and April 2020 compared to same period in 2019. Rapid assessments conducted at global levels and in the Eastern and Southern Africa Region on the impact of COVID-19 on continuity of essential health services confirm the disruption in provision of essential routine services, including immunization. Although most countries adopted and implemented WHO guidelines for maintaining routine immunization [3] surveys highlighted disruption of both supply and demand. On the supply side, medical personnel who normally provide essential primary health services were deployed to respond to COVID-19; frontline health workers hesitated to provide services because of shortages of personal protective equipment (PPEs), additionally, some health facilities were converted into COVID-19 isolation and treatment centres. Global supply chains for vaccines and vaccination supplies faced substantial delays in delivery times due to disruptions in transportation, movement restriction and increase in air freight costs.

The movement restrictions and the costs of accessing vaccination reduced the utilization of immunization services. Misinformation and rumours related to infection and vaccine trials have increased and constitute a barrier. Postponement and cancelation of supplementary immunization activities (SIAs) in Eastern and Southern Africa because of the COVID-19 pandemic have increased risks of vaccine-preventable disease outbreaks and reduced population´ immunity. At least 56 million targeted individuals in 2020 have missed at least one vaccination [4].

### We must act now to build stronger immunization programs

#### Review the immunization delivery systems resilience

Immunization outreach services are an entry point for Primary Health Care (PHC) services and an opportunity for a comprehensive package of basic services. It has been documented that the number of deaths caused by malaria, measles, tuberculosis, and HIV/AIDS, attributable to health system failures during Ebola Virus Disease (EVD) epidemic in west Africa (2014-2016) outnumbered the direct deaths from EVD [5]. Strategies to maintain equitable access to essential health services must be regarded as a critical part of the response to the COVID-19 pandemic.

#### Rebuild trust and create demand for immunization services

At onset of the spread of the COVID-19 pandemic, countries had to tackle the unprecedented level of misinformation about the disease, which caused public misunderstanding, fear and decline in utilization of essential health services including immunization. To rebuild trust and increase acceptance and demand for vaccination services mass media engagement and community engagement are essential [6].

#### Provide equitable access to essential services to all

Immunizations are typically offered free-of-charge as part of the national Universal Health Coverage package and programs have an obligation to ensure access to vaccinations for all. The strategies employed over the last decade to improve coverage and equity have not been effective overcoming the barriers to vaccination faced by children from poor, uneducated and marginalized households.

The need to catch-up missed vaccination services post-COVID offers an opportunity to build back more equitable vaccination services. The idea of providing fair services that reach all children must become mainstreamed in the planning and monitoring of immunization services. This demands better understanding of who the under-vaccinated groups and the zero-dose children are and where they live, and the implementation of effective pro-equity strategies for overcoming the barriers that prevent parents from vaccinating their children. Programs must improve monitoring of trends in inequality and the impact of pro-equity strategies as closely as they monitor changes in overall coverage. The Equity Reference Group has guidance on the most critical determinants of low vaccination uptake and the most promising pro-equity strategies for improving access [7]. Overcoming gender-related barriers to vaccination related to women´s position in society has emerged as a central theme in pro-equity programming and an important determinant of vaccination uptake in all contexts.

Other contexts where programs should expect high numbers of zero-dose and partially vaccinated children are urban poor communities, nomadic populations and families in remote rural areas, in areas where health services and families are affected by insecurity and conflict, including among internally displaced people and refugees. It is important that national programs identify and quantify the number of missed children in each of these contexts (and any other country-specific contexts) and design targeted strategies for reducing access to vaccines. The start for that process is to conduct a Coverage and Equity Assessment of the vaccination services, set targets, prioritize interventions and monitor outcome (reduction in inequalities) as well as process indicators (implementation of pro-equity strategies). It is important to consider structural inequalities in the health system such as the health workers and health facility ratio per population, average distances to services and cold chain capabilities as these often are important determinants of inequities in access to services.

The COVID-19 pandemic has caused major disruptions in immunization programmes: measles, polio and other preventive campaigns were suspended for several months and utilization of routine immunization services was reduced due to access challenges. Prior to the COVID-19 pandemic, over 6 million children in the region missed vaccination annually. COVID-19 impacts have uncovered gaps and challenges in the performance of the immunization programme. Catching-up on lost ground will require building better systems and enhancing the equity agenda for primary health care.
